# Neurocognitive profile of the adult population living with type 1 diabetes mellitus: a matched case-control cross-sectional study of metabolism and memory

**DOI:** 10.3389/fendo.2025.1660384

**Published:** 2025-10-28

**Authors:** Sultan Ayoub Meo, Metib Alotaibi, Narmeen Shaikh, Abdullah M. Alguwaihes

**Affiliations:** ^1^ Department of Physiology, College of Medicine, King Saud University, Riyadh, Saudi Arabia; ^2^ Department of Medicine (Diabetes Unit), College of Medicine, King Saud University, Riyadh, Saudi Arabia; ^3^ College of Medicine, King Saud University, Riyadh, Saudi Arabia; ^4^ Department of Medicine (Division of Endocrinology), College of Medicine, King Saud University, Riyadh, Saudi Arabia

**Keywords:** type 1 diabetes mellitus, cognitive function, adults, HbA1c, CANTAB

## Abstract

**Background:**

Diabetes Mellitus (DM) is a chronic, debilitating condition that causes numerous long-lasting complications. The cognitive health of people with DM is crucial for ensuring holistic development, academic success, and participation in daily life activities. This study aimed to assess the neurocognitive profile and the impact of HbA1c levels and disease duration on the cognitive profile of adults living with type 1 diabetes mellitus (T1DM).

**Methods:**

A total of 108 adults (54 T1DM, and 54 controls) were recruited, matched for age, gender, ethnicity, education, and Body Mass Index (BMI). The cognitive functions were measured using the Cambridge Neuropsychological Test Automated Battery (CANTAB). Four different tests were selected to assess the cognitive functions related to executive function, reaction time, visual memory, and spatial working memory in people with T1DM and their matched control group. Further analysis within the diabetic group was conducted based on HbA1c levels, disease duration, and the presence of hypoglycemic symptoms.

**Results:**

The Attention Switching Task parameters (AST mean correct latency, AST mean correct latency-congruent, AST Mean correct latency-incongruent) show that people with T1DM took significantly longer to respond to the task than the control group (p < 0.001). Moreover, the T1DM group exhibited significantly longer response times in the choice reaction time task (p < 0.001). Additionally, people with T1DM had significantly lower scores in the Pattern Recognition Memory task, suggesting impaired visual memory performance compared to the control group (p < 0.001). Furthermore, the diabetic group made significantly more errors in the spatial working memory task SWM (p<0.001), indicating difficulties in remembering and using spatial information. However, HbA1c levels, disease duration, and the presence or absence of hypoglycemic symptoms in the preceding month among the diabetic group were not associated with measurable differences in any of the cognitive tests.

**Conclusion:**

Cognitive performance was significantly impaired among adults with T1DM. T1DM participants showed slower processing speed, weaker executive functioning, and poorer memory performance compared to well-matched healthy controls. The study’s findings underscore the importance of glycemic management in adults with T1DM. These findings support physicians and policymakers in mitigating cognitive deficits among adults with T1DM.

## 1 Introduction

Diabetes mellitus (DM) has become a significant global public health issue with an increasing prevalence across the globe. DM affects all age groups, both genders, the urban and rural population, as well as developing and developed nations (1, 2). The International Diabetes Federation (IDF) indicates that worldwide, 590 million people, or 11.1% of the global population, have diabetes mellitus. The incidence of diabetes is projected to rise to about 853 million (13%) by the year 2050. Moreover, an estimated 635 million adults are living with impaired glucose tolerance (12%), and 252 million adults are unaware that they have the condition (3).

Type 2 diabetes mellitus (T2DM) is the predominant form, representing more than 90% of all diabetes cases globally, whereas type 1 diabetes mellitus (T1DM) accounts for approximately 10%. T1DM is most commonly diagnosed in school-aged and early university-aged children, adolescents and young adults. The epidemiological trends of type 1 diabetes mellitus (T1DM) are variable at both regional and global levels. Approximately 9.5 million people live with type 1 diabetes mellitus (T1DM) globally, compared to 8.4 million in 2021, representing a 13% increase. About 1.0 million of these people are aged 0-14, and 0.8 million are aged 15-19 years. The prevalence is markedly increased in low-income states, rising from 1.8 million in 2021 to 2.1 million by mid-year 2025, a 20% increase in the occurrence of T1DM. The estimated population with T1DM for 2040 is 14.7 million, with a life expectancy for a 10-year-old person with T1DM in 2025 varying between states, ranging from 6 to 66 years (3–5).

DM causes serious and long-lasting complications with huge disabilities and deaths. DM caused over 3.4 million deaths in 2024. This corresponds to 9.3% of global deaths from all causes. Moreover, over USD 1 trillion was spent on diabetes in 2024. This accounts for 12% of global health expenditure (3–5).

The steepest growth in diabetes prevalence has been observed in “low and middle-income nations across Southeast Asia (Malaysia); South Asia (Pakistan), the Middle East, North Africa (Egypt), and Latin America and the Caribbean (3)”. Global literature primarily emphasizes the impact of DM on multiple organ systems. However, the studies on cognitive functions in adults with T1DM have received comparatively less attention. Therefore, this study aims to assess the cognitive profile of adults with type 1 diabetes mellitus (T1DM) and to investigate the impact of HbA1C and diabetes duration on cognitive performance in adults with T1DM.

## 2 Subjects and methods

This “matched case-control cross-sectional study was conducted in the Physiology Department and the Diabetes Centre, College of Medicine, King Saud University, Riyadh, Saudi Arabia, during the period November 2023 to August 2024”.

### 2.1 Inclusion and exclusion criteria

The study population consisted of adults with T1DM who visited the university diabetes center, King Saud University Hospitals. The control group comprised individuals with no known history of diabetes mellitus. The control group was recruited from attendants who visited the hospital. The control group consisted of non-diabetic individuals with similar ages, genders, and ethnicities. The individual must be at least 19 years old, of Saudi ethnicity, able to communicate in Arabic or English, and hold at least a high school diploma. For the T1D group, an additional criterion was that they must have been clinically diagnosed with type 1 diabetes mellitus (T1DM).

Exclusion criteria included the following: T1DM and control group younger than nineteen; history of mental confusion, anxiety, depression, sleep deprivation, visual problems, psychiatric disorders, neuropathy, retinopathy, cerebrovascular diseases; participants who smoked cigarettes, shisha, or other addictive substances were excluded from the study. For the control group, an additional exclusion criterion was that if they had been diagnosed with type 1 or type 2 diabetes, they used cigarettes, shisha, or had a clinical history of any debilitating diseases, they were excluded from the study.

### 2.2 Data collection

We used convenience sampling to recruit adults with “T1DM who visited the Diabetes Unit at King Saud University Hospitals, Riyadh, Saudi Arabia. The T1DM group was matched with the control group for the same age, gender, ethnicity, weight, height, BMI, and level of education” to achieve the appropriate study outcomes for cognitive functions and minimize the study bias factors. The minimum sample size for this study was determined using the power formula. A sample size of 64 participants (32 T1D and 32 controls) was sufficient to attain 95% confidence with a 5% margin of error. However, for this study, we recruited an adequate number of participants, comprising 108 volunteers (54 with T1DM and 54 controls).

In this study, 50 T1DM participants were on Basal-bolus insulin therapy with a continuous glucose monitoring (CGM) sensor, and 4 were on sensor-augmented pump. For the diabetic group, the following data were collected from files: age, gender, height, weight, BMI, HbA1C, duration of diabetes, and history of other comorbidities. A recent history of hypoglycaemic symptoms and diabetic ketoacidosis (DKA) was also collected from the medical records. The T1DM group, who received a confirmed diagnosis of DKA from medical professionals, was included under this definition. Participants were also asked about smoking history and the presence of any of the comorbidities. For HbA1C, we extracted the values from the previous year and got an average for everyone. HbA1C was “further categorized into two groups for the analysis: HbA1C < 8% and HbA1C ≥ 8%. Duration of diabetes was categorized into three groups: < 10 years, 10-19 years, and 20 or more years”. Hypoglycaemic symptoms in the last month were categorized as yes or no. DKA symptoms in the previous year were categorized as yes or no. For the control group, the following data were collected from the participants: age, gender, height, weight, BMI, smoking history, and information regarding their history of diabetes and/or any of the comorbidities mentioned in the exclusion criteria above. Everyone in both groups was assigned a code. Apart from recording the hospital file number for the diabetic group to access their hospital records for HbA1C levels, no other identifying information was recorded. For the control group, no identifying information was collected.

### 2.3 Cognitive performance testing:

Cognitive performance testing was conducted using the “Cambridge Neuropsychological Test Automated Battery (CANTAB) (6)”. The tasks were based on interactions with a touchscreen computer. For our study, the tests were administered by a trained assistant. The test procedure was explained to the study participants, who were seated comfortably with a laptop on the table. They were informed that the procedure would take 25 to 30 minutes, and instructions were repeated if needed. Four tests were chosen for our study. Each test has been described in detail in one of the articles (6). Below are a summary of the four tests and the outcome of the measure that was chosen for each:

#### 2.3.1 Attention switching task

This test assesses frontal lobe and executive function (6). It assesses the participant’s ability to shift focus between the direction or position of an arrow displayed on the screen. The AST has several outcome parameters, from which we chose “AST Mean correct latency, AST Mean correct latency (congruent), and AST Mean correct latency (incongruent)”. The unit for all tests was milliseconds (ms). If all three latencies are low, the participant is both quick at basic responses and efficient at switching rules.

#### 2.3.2 Choice reaction time

CRT is one of the attention tests offered by CANTAB, which assesses individuals’ alertness and motor speed (6). Participants must press buttons on a press pad in response to the stimulus shown on the screen. We recorded CRT mean correct latency (ms) and CRT percent correct trials (%) from all available outcome measures. Lower latencies denote quicker alertness and motor execution, while higher percentages indicate greater response accuracy.

#### 2.3.3 Pattern recognition memory

This is a test for visual memory (6). Participants were presented with a series of visual patterns that they should be able to recall later. We extracted PRM percent correct (%) values for the outcome measure. Higher percentages signify better visual episodic memory.

#### 2.3.4 Spatial working memory

This test measures the ability to recall spatial information and remembered items in working memory. We chose SWM between errors and SWM strategy as our outcome measures. SWM Between-errors shows the number of times a participant revisits a box that has already yielded a token within the same trial; fewer errors represent more accurate spatial working-memory maintenance. A low score in the SWM strategy indicates a more efficient, systematic search strategy (better executive planning).

#### 2.3.5 Statistical analysis

All statistical analyses were conducted using SPSS Statistics version 29. Continuous variables are summarized as mean ± SD and categorical variables as counts and percentages. Every cognitive outcome distribution was screened for normality by visual inspection of its histogram and normal Q–Q plot. The first analysis compared cognitive-test scores between T1DM participants and their matched non-diabetic controls using a paired samples t-test. We performed comparisons within the diabetes cohort after stratifying by HbA1c (<8% vs.≥8%) and duration of disease (<10 years, 10–19 years, ≥20 years). The presence or absence of hypoglycaemic episodes in the previous month was assessed using an independent-samples t-test, or an ANOVA for three-category factors. Diabetic ketoacidosis was not analyzed because only two participants reported an episode during the past year. The p-value of < 0.05 was considered significant.

##### 2.3.5.1 Sensitivity analysis

To explore potential non-linear associations between glycaemic control or diabetes duration and cognitive performance, a sensitivity analysis was conducted. Participants were stratified into tertiles based on HbA1c levels and diabetes duration, respectively, with each tertile group representing approximately one-third of the sample (low, mid, and high).

One-way analysis of variance (ANOVA) was used to compare cognitive performance across tertiles for each outcome, assuming that assumptions of normality and homogeneity of variances were met. When significant differences were observed, *post-hoc* pairwise comparisons were conducted using Tukey’s HSD test.

## 3 Results

Our study had 108 participants (54 T1DM and 54 controls). Each diabetic individual was matched with a control group member (**Table 1**) based on age, height, weight, and BMI. The mean age of our diabetic group was 33.30 years, and the mean BMI was 27.56 kg/m2. Approximately 55.6% of the group consisted of males, and the remaining 44.4% were females. The mean HbA1C of the diabetic group was 7.66%. The majority of our diabetic group (29.6%) had diabetes for 10-19 years. 22% of people with diabetes experienced hypoglycaemic symptoms in the last month, while only 3.7% had clinically diagnosed DKA in the previous year. Other information about the diabetic group’s kidney functions, lipid profile and vitamin profile is also provided in **Table 1**.

**Table 1:** Descriptive characteristics of diabetic and control groups.

Variables	Control group (n=54)	Diabetic group (n=54)	P-value
	Mean ± SD	Mean ± SD	
Age (years)	34.19 ± 9.503	33.30 ± 9.33	0.62
Height (cm)	163.76 ± 9.08	162.08 ± 9.83	0.36
Weight (kg)	72.34 ± 13.23	72.58 ± 15.78	0.93
BMI (kg/m2)	26.96 ± 4.43	27.56 ± 5.26	0.54
HbA1C (%)	–	7.66 ± 1.08	N/A
Lipid Profile			
Total Cholesterol (mmol/L)	–	4.88 ± 1.192	N/A
LDL (mmol/L)	–	2.85 ± 1.104	N/A
HDL (mmol/L)	–	1.55 ± 0.353	N/A
Triglycerides (mmol/L)	–	1.03 ± 0.814	N/A
Kidney Profile			
Serum Creatinine (mg/dL)	–	73.09 ± 35.051	N/A
BUN (mg/dL)	–	4.14 ± 2.677	N/A
Others			
Vit D (ng/mL)	–	61.81 ± 31.450	N/A
B12 (pg/mL)	–	334.20 ± 126.574	N/A
	N (%)	N (%)	
Gender			
Male	24 (44.4)	24 (44.4)	N/A
Female	30 (55.6)	30 (55.6)	N/A
Duration of T1DM (years)			
< 10 years	–	11 (20.4)	N/A
10-19 years	–	16 (29.6)	N/A
20-29 years	–	15 (27.8)	N/A
≥ 30 years	–	12 (22.2)	N/A
Hypoglycaemic Symptoms during the Last Month			
No	–	32 (59.3)	N/A
Yes	–	22 (40.7)	N/A
Diabetic Ketoacidosis (DKA) during the last year			
No	–	52 (96.3)	N/A
Yes	–	2 (3.7)	N/A

N/A, Not applicable

### 3.1 Cognitive function tests

**Figure 1:**
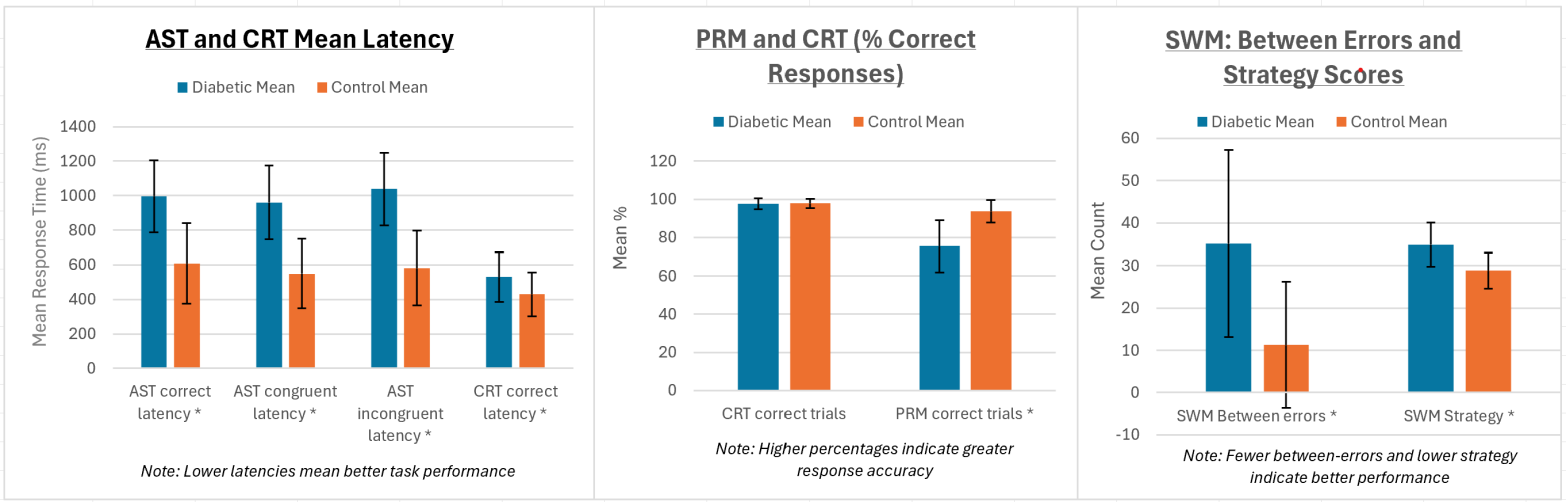
AST, CRT, PRM, and SWM performances compared between diabetic and control groups. Error bars represent standard deviations. All tests with (*) represent statistically significant differences (p = 0.001) between the groups.

We first established the comparison of test parameters between the diabetic and control groups (**Table 2**). The results of the Attention Switching Task parameters (AST mean correct latency, AST mean correct latency-congruent, and AST Mean correct latency-incongruent) show that individuals with diabetes took significantly longer to correctly respond to the task than the control group (p < 0.001). Similarly, the diabetic group exhibited significantly longer response times in the choice reaction time task (p < 0.001), which measures the speed of decision-making. However, the insignificant difference in the CRT per cent correct trials between the diabetic and control groups (p = 0.66) suggests that, although the diabetic group was slower, their accuracy was not significantly affected. The diabetic group had significantly lower scores in the Pattern Recognition Memory task, indicating impaired visual memory performance compared to the control group (p < 0.001). Finally, the SWM between error averages shows that the diabetic group made significantly more errors in the spatial working memory task (p < 0.001), indicating difficulties in remembering and using spatial information. The high SWM strategy mean value for the diabetic group showed significantly worse strategy use in spatial working memory tasks (p < 0.001). Overall, the results across multiple cognitive tasks suggest that individuals with T1DM have significant impairments in various cognitive functions, including processing speed, memory, and executive function, compared to a matched control group (**Table 2**; **Figure 1**).

**Table 2:** Cognitive functions of people with type 1 diabetes mellitus and matched controls.

Tests	Diabetic group (mean ± SD)	Control group (mean ± SD)	P-value
AST Mean correct latency (ms)	995.77 ± 210.01	607.76 ± 231.17	0.001*
AST Mean correct latency (congruent) (ms)	960.49 ± 214.57	548.95 ± 201.12	0.001*
AST Mean correct latency (incongruent) (ms)	1037.55 ± 211.75	580.37 ± 216.48	0.001*
CRT Mean correct latency (ms)	529.93 ± 143.10	429.86 ± 126.94	0.001*
CRT Percent correct trials (%)	97.50 ± 2.83	97.74 ± 2.36	0.66
PRM Percent correct trials (%)	75.41 ± 13.65	93.60 ± 5.84	0.001*
SWM Between errors	35.14 ± 22.03	11.25 ± 14.92	0.001*
SWM Strategy	34.89 ± 5.22	28.79 ± 4.26	0.001*

AST, Attention switching task; CRT, Choice reaction time; PRM, Pattern recognition memory; SWM, Special working memory; ms, millisecond. *Significance level.

### 3.2 Cognitive function tests analysis based on HbA1C levels within the diabetic group

Diabetic participants were divided into two glycaemic-control groups: HbA1c < 8% (n = 38) and HbA1c ≥ 8% (n = 16). **Table 3** summarizes the mean ± SD for each CANTAB outcome and their p-values. None of the eight cognitive indices differed significantly between the two HbA1c groups.

**Table 3:** Cognitive functions of people with type 1 diabetes mellitus categorized by HbA1C level.

Tests	HbA1C < 8% mean ± SD (n=38)	HbA1C ≥ 8% mean ± SD (n=16)	p-value*
AST Mean correct latency (ms)	988.84 ± 213.26	1012.23 ± 207.94	0.711
AST Mean correct latency (congruent) (ms)	954.08 ± 218.01	975.72 ± 212.33	0.737
AST Mean correct latency (incongruent) (ms)	1029.30 ± 214.10	1057.13 ± 211.60	0.663
CRT Mean correct latency (ms)	507.51 ± 126.12	593.44 ± 173.51	0.136
CRT Percent correct trials (%)	97.79 ± 2.78	96.67 ± 2.93	0.261
PRM Percent correct trails (%)	76.58 ± 14.61	71.67 ± 9.86	0.169
SWM Between errors	35.44 ± 21.68	27.67 ± 17.17	0.287
SWM Strategy	33.93 ± 5.88	33.56 ± 6.00	0.874

AST, Attention switching task; CRT, Choice reaction time; PRM, Pattern recognition memory; SWM, Special working memory; ms, millisecond. *Group comparisons across HbA1c categories were evaluated with Welch’s independent t-test.

For the Attention-Switching Task (AST), the two HbA1c groups performed almost identically: mean correct latencies for the overall, congruent, and incongruent conditions differed by less than 30 ms, and none of the comparisons were statistically significant (p = 0.711, 0.737 and 0.663). Because higher latency reflects slower mental processing, these results suggest that switching attention was equally rapid in individuals with T1DM, regardless of HbA1c levels below and above 8%. For the Choice-Reaction-Time (CRT) test, although the ≥ 8% group was numerically slower (593 ± 174 ms vs 508 ± 126 ms) and marginally less accurate (96.7 ± 2.9% vs 97.8 ± 2.8%), neither latency nor accuracy reached significance (p = 0.136 and 0.261). Pattern-recognition memory showed a slight, non-significant advantage for the better-controlled group: participants with HbA1c < 8% recalled 76.6 ± 14.6% of items compared with 71.7 ± 9.9% in the ≥ 8% group (higher percentages indicate better memory), but the results did not reach statistical significance. Finally, Spatial Working-Memory (SWM) measures showed no difference between groups: the higher-HbA1c participants made slightly fewer between-search errors (27.7 ± 17.2 vs 35.4 ± 21.7, p = 0.287) and achieved virtually identical strategy scores (33.6 ± 6.0 vs 33.9 ± 5.9, p = 0.874), but the observed variations were small and non-significant.

These analyses indicate that categorizing the T1DM group by an HbA1c threshold of 8% did not reveal measurable differences in attention switching, reaction speed, memory accuracy, or spatial working memory performance.

### 3.3 Cognitive function tests analysis based on duration of diabetes

**Table 4** compares mean scores on eight CANTAB outcomes across three durations of diabetes categories: < 10 years (n = 11), 10–19 years (n = 16) and ≥ 20 years (n = 27). Analysis showed no statistically significant differences for any variable. AST scores were remarkably similar across groups: mean correct latency was 1,008 ± 260 ms in the < 10-year group, 947 ± 158 mms in the 10–19-year group, and 1,019 ± 218 ms after ≥ 20 years (p = 0.454). The same pattern held for both the congruent and incongruent conditions, indicating that attentional-shifting speed was unaffected by disease duration. For CRT, the mean latency tended to be lowest in the 10–19-year group (485 ± 101 ms) and highest in the ≥ 20-year group (569 ± 155 ms), but the difference did not reach significance (p = 0.172). Accuracy was identical across the three categories, and the overall per cent-correct score difference was not significant. For the SWM test, the number of between-search errors increased modestly with longer disease duration; however, this trend did not achieve significance. Overall, our ANOVA analysis revealed that, within this cohort, cognitive speed, accuracy, memory, and spatial working memory measures did not differ significantly according to the duration of disease (**Table 4**).

**Table 4:** Cognitive function test categorized by duration of diabetes within the diabetic group.

Tests	< 10 years (n= 11) mean ± SD	10-19 years (n= 16), (mean ± SD)	≥ 20 years (n=27), (mean ± SD)	p-value*
AST Mean correct latency (ms)	1008.16 ± 260.44	947.28 ± 157.85	1019.47 ± 217.59	0.454
AST Mean correct latency (congruent) (ms)	969.79 ± 252.67	911.05 ± 158.82	985.99 ± 229.19	0.451
AST Mean correct latency (incongruent) (ms)	1059.42 ± 281.56	985.44 ± 156.89	1059.52 ± 210.56	0.409
CRT Mean correct latency (ms)	511.24 ± 166.44	485.45 ± 101.14	569.07 ± 155.23	0.172
CRT Percent correct trials (%)	97.00 ± 3.63	97.56 ± 2.50	97.63 ± 2.87	0.907
PRM Percent correct trails (%)	69.99 ± 15.19	80.83 ± 11.33	73.92 ± 13.36	0.114
SWM Between errors	24.40 ± 18.37	27.92 ± 21.91	40.06 ± 19.23	0.184
SWM Strategy	32.80 ± 7.69	33.15 ± 5.46	34.61 ± 5.79	0.755

AST, Attention switching task; CRT, Choice reaction time; PRM, Pattern recognition memory; SWM, Special working memory; ms, millisecond.

### 3.4 Cognitive function tests analysis based on history of hypoglycaemia symptoms

Within the diabetic cohort, we compared participants who did not report hypoglycaemic symptoms during the preceding month (n=32) with those who did report such symptoms (n=22). As summarized in **Table 5**, no cognitive measure differed significantly between the two subgroups for the AST. Mean correct latencies did not differ significantly between participants with hypoglycemic symptoms (947 ± 170 ms) and those without symptoms (1,029 ± 230 ms; p= 0.138). This non-significant pattern was also evident for both the congruent and incongruent trials. The groups were identical in mean reaction speed for CRT and did not differ in CRT accuracy either. The PRM accuracy was slightly higher in the hypoglycaemia group (77.4 ± 13.6%) than in the no-symptom group (73.7 ± 13.5%), but this difference was not significant. For the Spatial Working Memory task, there was a slight, non-significant performance advantage for participants without recent hypoglycaemic symptoms. They made slightly fewer between-search errors (32.7 ± 22.9 vs. 34.7 ± 17.9) and employed a marginally more efficient strategy, as reflected by a lower strategy score (33.0 ± 6.9 vs. 35.0 ± 3.8). Although neither difference reached statistical significance (**Table 5**). Overall, a recent history of hypoglycemic symptoms was not associated with measurable differences in attention, reaction time, visual memory, or spatial working memory performance.

**Table 5:** Cognitive functions with type 1 diabetes mellitus categorized by history of hypoglycaemic symptoms in the last one month.

Tests	Hypoglycaemic symptoms in last 1 month - NO (n= 32) (mean ± SD)	Hypoglycaemic symptoms in last 1 month - YES (n= 22) (mean ± SD)	p-value*
AST Mean correct latency (ms)	1029.26 ± 229.99	947.07 ± 170.39	0.138
AST Mean correct latency (congruent) (ms)	993.96 ± 234.10	911.80 ± 176.38	0.148
AST Mean correct latency (incongruent) (ms)	1069.48 ± 232.73	991.10 ± 171.45	0.160
CRT Mean correct latency (ms)	528.24 ± 167.55	532.31 ± 103.14	0.919
CRT Percent correct trials (%)	97.04 ± 3.02	98.16 ± 2.48	0.175
PRM Percent correct trails (%)	73.66 ± 13.45	77.38 ± 13.60	0.336
SWM Between errors	32.67 ± 22.89	34.67 ± 17.87	0.770
SWM Strategy	33.00 ± 6.88	35.00 ± 3.84	0.274

AST, Attention switching task; CRT, Choice reaction time; PRM, Pattern recognition memory; SWM, Special working memory; ms, millisecond.

### 3.5 Cognitive performance across tertiles

No statistically significant differences were observed in cognitive test performance across the three HbA1c tertiles (all p-values > 0.05) and diabetes duration tertiles (all p-values > 0.05). Descriptive statistics (mean ± SD), ANOVA, and *post-hoc* results for both are summarized in **Supplementary Tables S1 and S2**.

## 4 Discussion

Worldwide, T1DM is increasingly recognized not only for its classic systemic complications but also for its impact on the central nervous system and cognition. T1DM has an association with decreased cognitive function (7, 8). In the present study, we found that adults with T1DM had significantly poorer cognitive performance than their matched non-diabetic controls across multiple domains, including attention switching speed, reaction times, visual memory, and spatial working memory.

The previous studies have reported that individuals with T1DM exhibit reduced cognitive performance compared to their non-diabetic controls (9–14). For example, a meta-analysis by Brands et al. (15) found significantly lower performance in the T1DM group compared with nondiabetic controls across several domains, including information processing speed, psychomotor efficiency, attention, and cognitive flexibility. Tonoli et al. (16) reported in an updated meta-analysis that there was a mild to modest decrease in cognitive performance in the T1DM group compared to non-diabetic controls. Adults with T1DM performed worse than controls on executive function, memory, and motor speed. The overall magnitude of cognitive disadvantage in T1DM is considered mild to moderate. Our findings reinforce this consensus. We observed significant differences between the diabetic and the control group in reaction time-based measures of processing speed and tasks requiring executive planning, visual memory, and working memory.

A recent systematic review and meta-analysis reported that across several studies of adults with T1DM, participants with poorer glycaemic control generally scored lower on overall IQ tests than those with better control. In contrast, the differences for verbal IQ, memory, and attention were not significant (17). Another study found that poor glycaemic control in persons with diabetes was linked with cognitive impairment (18). In another study, authors followed a large group of T1DM patients, and it was found that higher glycated hemoglobin values were associated with moderate declines in motor speed and psychomotor efficiency; however, no other cognitive domain was affected (19). Research has also shown that having a 14-year average HbA1c < 7.5% tripled the odds of cognitive impairment (11). The literature also demonstrates reduced cognitive performance among children and adolescents with T1DM, which was associated with high HbA1C and disease duration (1).

In contrast, a study’s findings suggested that chronic hyperglycaemia does not have an independent effect on cognitive change; however, it may moderate the relationship between retinopathy and cognitive change (20). In our results, none of the cognitive domains showed any significant associations between high and low HbA1c categories. An explanation for our results not detecting an association is that we summarized glycaemic control with a single, annualized mean HbA1c. A one-year snapshot can miss the cumulative hyperglycaemic exposure that unfolds over decades. Johnston et al. (20) support this, as their results show that long-term HbA1c values are more meaningful than isolated HbA1c snapshots, demonstrating a stronger association with lifetime changes in cognitive function. Our negative result may therefore reflect the crudeness of a single annualized HbA1c average, and detecting subtle cerebral effects may require more detailed indicators of long-term hyperglycaemia and day-to-day glucose fluctuations.

Similarly, we did not observe a relationship between diabetes duration and cognitive performance in this adult sample. One study found that longer diabetes duration (≥5 vs. <5 years) was significantly associated with incident cognitive impairment (18). Brismar et al. (21) found that people with long disease duration and younger age of T1DM onset scored lower in multiple domains (psychomotor speed, memory, attention, working memory, etc.), suggesting that those who live with T1DM from an early age may accumulate more cognitive deficits over time. In contrast, Ryan et al. (13) reported that the duration of the disease significantly predicted the decline in psychomotor speed. One plausible explanation for our results, which showed non-significant associations, is that the amount of duration is less critical than the quality of that duration, meaning that the more important factor affecting the brain is whether long-term T1DM has led to microvascular complications or other comorbidities, rather than the duration itself. This concept is supported by a systematic review and meta-analysis that discusses articles not showing a consistent pattern regarding the direct impact of disease duration on cognition and suggests that the effects of juvenile onset and the occurrence of diabetes complications may have a greater impact (15).

Biessels et al. (22), noted that diabetes-related cognitive decline typically appears either in childhood, when the brain is still maturing, or in later life, when neurodegeneration accelerates. Outside these periods, it is chiefly observed in people with diabetes who already have significant micro- or macrovascular complications. In our study, the lack of a duration effect, coupled with the clear group difference versus controls, suggests that most T1DM participants, whether 10- or 30-years post-diagnosis, had already incurred a mild cognitive impact, but additional years with the disease did not dramatically exacerbate that impact in the absence of other factors. Moreover, improvements in modern diabetes care, such as intensive insulin regimens, glucose-sensing technology, and education programs introduced over the past two decades, may have mitigated the cumulative cerebral impact that earlier cohorts experienced.

We also examined the influence of the presence of hypoglycaemia in the previous month and found no relationship between recent hypoglycaemic episodes and cognitive performance. Prior evidence remains inconsistent; some studies found no relationship between hypoglycaemic episodes and cognitive decline (15, 21), whereas others report that hypoglycaemia does impair cognition, with the most noticeable impact on basic rather than complex tasks (23). Transient mild to moderate hypoglycaemia (e.g., episodes recognized and self-treated, without seizures or loss of consciousness) has short-term cognitive effects (such as reduced attention or slowed response during the episode) but does not typically produce lasting cognitive deficits in adults with type 1 diabetes mellitus (T1DM) (24, 25). This makes clinical sense, given the brain’s ability to recover from brief glucose dips. The concern in the literature has always been more about severe hypoglycaemia episodes, leading to unconsciousness or seizure, as a potential cause of neuronal injury, and even then, the research findings can be mixed. It has been reported that in adults, executive function and memory were both significantly affected by severe hypoglycaemia (16). A study found that T1DM participants with incident severe hypoglycaemic events performed worse on overall cognitive functioning and information processing speed (26). Another study found that an episode of severe hypoglycaemia in the past year was associated with poorer cognitive test scores (27). In contrast, evidence also showed that among young people with T1DM, repeated bouts of severe hypoglycaemia alone did not alter brain structure or function (28).

T1DM affects the microstructural and cognitive functions of the brain, and brain volume is reduced in people with diabetes. T1DM may impair cognitive performance due to changes in brain microstructures (29). It causes white matter alterations in the thalamocortical tract, while impairing its role in sensory inputs from the thalamus to the cerebral cortex (30). The literature highlights reduced volumes of white and grey matter in youth with T1DM (31).

### 4.1 Study strengths and limitations:

Similar to other studies, this research has some limitations. Participants were recruited from a single tertiary center, using convenience sampling, specifically from the Saudi population, and subgroup sizes were small. Glycemic control was assessed only by the mean HbA1c over the previous year, and self-reported episodes of hypoglycemia. The cross-sectional design cannot establish causality. All these factors may limit the generalizability of our study findings. Despite these limitations, this study has several strengths. Participants were matched based on their age, gender, weight, height, BMI, ethnicity, and educational status. The cognitive assessment was conducted using a reliable device and a well-validated tool, the CANTAB battery. Such studies are needed for a better understanding of physicians about the impact of diabetes on cognitive functions.

## 5 Conclusion and significance

This study adds to the growing body of evidence that T1DM in the adult population is associated with modest but significant deficits in multiple cognitive domains. Our T1DM participants showed slower processing speed, weaker executive functioning, and poorer memory performance compared to well-matched healthy controls. Importantly, we found that these cognitive differences were not explained by variations in current glycemic control, disease duration, or recent mild hypoglycemia, which highlights the role of long-term pathogenic processes (e.g., cumulative hyperglycemic exposure or microvascular brain changes) rather than transient metabolic fluctuations. Our results show that even in otherwise healthy middle-aged adults with T1DM, there may be subtle cognitive vulnerabilities. From a clinical perspective, such cognitive inefficiencies, although not overtly disabling, could impact diabetes self-management behaviors that rely on quick thinking, working memory, and planning. Recognizing these challenges is essential, and it may be beneficial to incorporate cognitive screening or support (such as memory aids or simplified treatment regimens) for people who struggle with diabetes management despite good education and motivation. Continued research in this area, including intervention trials and neuroimaging studies, will help clarify the mechanisms underlying cognitive changes associated with T1DM and determine the most effective strategies for mitigating their long-term effects on well-being.

## Data Availability

The raw data supporting the conclusions of this article will be made available by the authors with reasonable request.
