# Supplementary material for: Neurocognitive profile of the adult population living with type 1 diabetes mellitus: a matched case-control cross-sectional study of metabolism and memory
Source: Front Endocrinol (Lausanne). 2025 Oct 28;16:1660384. doi: 10.3389/fendo.2025.1660384 (PMC12602217; doi:10.3389/fendo.2025.1660384)
Supplement: Supplementary file 1 [file DataSheet1.pdf]

Supplementary Table S1: Cognitive Performance Across HbA1c Tertiles (Mean ± SD)

Cognitive Test	Tertile 1 Mean ± SD	Tertile 2 Mean ± SD	Tertile 3 Mean ± SD	p-value	Post-hoc Significant?
AST Mean Correct Latency	961.96 ± 262.90	1011.61 ± 168.39	1013.76 ± 195.47	0.712	None
AST Mean Correct Latency (Congruent)	918.97 ± 264.05	988.04 ± 176.59	974.47 ± 199.49	0.601	None
AST Mean Correct Latency (Incongruent)	1007.26 ± 267.61	1044.73 ± 163.78	1060.66 ± 199.40	0.747	None
AST Percent Correct Trials	85.56 ± 15.61	86.49 ± 11.86	80.90 ± 13.66	0.434	None
Choice Reaction Time	523.88 ± 140.40	500.08 ± 123.15	574.34 ± 167.17	0.348	None
CRT Percent Correct Trials	98.86 ± 1.66	97.11 ± 3.25	96.64 ± 2.87	0.087	None
Pattern Recognition Memory (PRM % Correct)	77.45 ± 15.31	74.77 ± 14.61	73.28 ± 10.53	0.668	None
SWM Between Errors	31.33 ± 22.16	38.85 ± 21.54	29.55 ± 18.48	0.510	None
SWM Strategy	31.50 ± 5.58	35.69 ± 5.82	34.18 ± 5.71	0.196	None

Supplementary Table S2: Cognitive Test Outcomes by Diabetes Duration Tertiles

Cognitive Test	Tertile 1 Mean ± SD	Tertile 2 Mean ± SD	Tertile 3 Mean ± SD	p-value	Post-hoc Significant?
AST Mean Correct Latency	983.7 ± 216.6	983.0 ± 172.7	1020.7 ± 244.5	.832	None
AST Mean Correct Latency (Congruent)	947.1 ± 209.5	941.5 ± 177.0	992.9 ± 258.1	.740	None
AST Mean Correct Latency (Incongruent)	1028.5 ± 235.1	1027.0 ± 171.9	1057.2 ± 233.2	.894	None
AST Percent Correct Trials	84.3 ± 17.4	86.4 ± 11.8	82.3 ± 11.8	.688	None
Choice Reaction Time	498.9 ± 141.5	504.8 ± 89.7	582.5 ± 175.2	.192	None

Choice Reaction Time – Percent Correct	97.6 ± 2.95	97.2 ± 2.51	97.7 ± 3.16	.884	None
Pattern Recognition Memory – Percent Correct	76.0 ± 14.9	76.7 ± 11.6	72.9 ± 14.2	.684	None
Spatial Working Memory – Between Errors	26.1 ± 18.4	32.0 ± 22.9	41.2 ± 19.2	.200	None
Spatial Working Memory – Strategy	33.0 ± 6.0	33.6 ± 5.1	34.8 ± 6.5	.758	None
